# Making literature reviews more ethical: a researcher and health sciences librarian collaborative process

**DOI:** 10.4155/fso.15.78

**Published:** 2015-11-01

**Authors:** Bejoy Thomas, Admasu Tachble, Delshani Peiris, Rebecca Malhi, Glenys Godlovitch, Yongtao Lin

**Affiliations:** 1 Department of Psychosocial & Rehabilitation Oncology, Tom Baker Cancer Centre, CancerContol Alberta, Alberta Health Services, Calgary, AB, T2S 3CI, Canada; 2 Department of Oncology, Cumming School of Medicine, University of Calgary, Calgary, AB, T2N 1N4, Canada; 3 Alberta Cancer Research Ethics Committee, CancerContol Alberta, Alberta Health Services, Calgary, AB, T2N 1N4, Canada; 4 Knowledge Resource Service, Knowledge Management Department, Tom Baker Cancer Centre, University of Calgary, Calgary, AB, T2N 1N4, Canada

**Keywords:** collaboration, ethics, evidence-based practice, health sciences librarian, literature search, malignant neoplasm, systematic reviews

## Abstract

**Background:**

With emphasis on evidence-based medical care, ‘evidence’ is often the result of literature reviews. Hence, the critical question, “are literature reviews comprehensive?”

**Aim:**

This study compares the literature generated by a researcher and a health sciences librarian (HSL).

**Methods:**

The Research Associate and the HSL conducted a parallel, segregated literature search on ‘patient-centered care’.

**Results:**

The Research Associate identified 215 manuscripts, and the HSL 129 manuscripts. Overlap was only 55 manuscripts. Differences in process and blind spots are discussed.

**Conclusion:**

To improve the quality of research outcomes, it seems prudent and ethical to have a synergistic collaboration between researchers and HSLs. Given that this is just one case study that has looked into the issue, further research is strongly encouraged.

## Abstract (lay abstract)

**Lay abstract:** Literature reviews are not just compilations of easily accessible research and should not be taken lightly. Literature reviews are critical to decision-making in clinical trials, medical care and directions of health systems. We wanted to see if researchers and health science librarians working in isolation would create a robust and balanced literature review. This case study showed that both approaches have shortcomings. We recommend that researcher and health science librarian must work together for the success of improving medical care.

**Figure 1.**
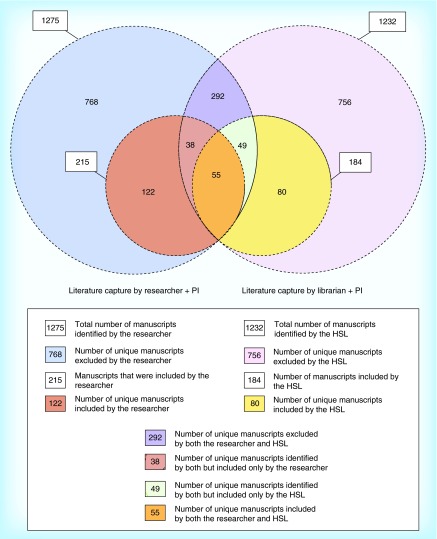
**Venn diagram indicating the total, overlap and unique literature capture between the two teams.** HSL: Health science librarian; PI: Principal investigator.

Research is cumulative in nature, building on prior studies on the topic of interest. Thus, it is important for the researcher to have robust understanding of the extant knowledge. A comprehensive literature review will describe the research concepts, ground critical appraisal of previously published studies and identify gaps or inconsistencies in the knowledge base, which merit further investigation. Reviewing the literature requires several skills, and is usually done in one of two ways. In the first approach, the researcher (or the research team) critically evaluates relevant information and effectively scans the literature for both breadth and depth of information, to the best of their ability [1]. Consequently, the researcher/team's capacity to locate and access appropriate studies influences the quality of the research reviewed [2].

The second option is to engage the services of a health sciences librarian (HSL) with in-depth knowledge of various evidence resources and the professional training to conduct comprehensive literature searches. The comparative advantage researchers have by working with a HSL in the research process is well documented [3–7]. Yet, the librarian often tends to have a subordinate role and the partnership could be summarized as ‘we (the researcher) gave them the key words, they had a few questions for us, and a week later we got the reference list’. Can the literature review process be optimized? In this case report, we compare the results of literature reviews conducted by a researcher and a HSL.

## Methods

### Review teams

Two separate teams were created for the literature search with a principal investigator (PI) taking the role of independent subject expert in each. One team was comprised of a PhD-trained research associate (RA; A Tachble) and the PI (B Thomas). The other team was comprised of the HSL (Master of Library Information Sciences; Y Lin) and the PI. The PI facilitated the process but did not share information between groups. Thus, both teams conducted parallel, segregated literature searches on the same topic.

### Literature review procedure

The literature review process was conducted in four steps: determine the topic of interest; define inclusion and exclusion criteria; compile the literature (total hits); and evaluate the literature to determine ‘relevant hits’ by relevance of the identified literature to the topic of interest (e.g., use of token keyword); a predefined context or setting ; the nature of each hit – is it original (clinical) research or a theoretical stance; and if the original research's methodology could facilitate a decision process to clinical practice (i.e., is process A better than, or equivalent or cost effective, among others compared with process B?)

## Results

In the first step, we determined the existing background information on the chosen topic – ‘patient-centered care’ – and began a broad-scale search to identify concepts and to create a provisional list of keywords: patient-centered care, family-centered care, cancer, malignant neoplasm, chronic disease(s), tertiary care, rural care and healthcare provider. For step two, we defined the inclusion criteria (articles published during 2000 and onwards, English language and print/electronic media access to full text, among others) and exclusion criteria (non-English language, personal communications, nonelectronic materials, publications dated prior to 2000 and pediatric/pediatric population) for the literature. The PI then provided these to the RA and the librarian.

The literature search (the third step) included searching through electronic research databases, conference proceedings, dissertation abstracts among others to identify published articles, reports and works in progress. Both teams accessed Cochrane, PsycInfo and MEDLINE data sources to locate published studies/literature addressing patient-centered care. In addition to these, the librarian also searched EMBASE, EBSCO Business Source Complete and Cumulative Index to Nursing and Allied Health Literature (CINAHL), ProQuest Dissertations and Thesis and Web of Science. The RA accessed PubMed and Google Scholar as well.

The literature reviews were evaluated and scored using the following metric:

Relevance of the identified literature to the topic of patient or family centeredness (10 points);
Context or setting: cancer (10 points) or chronic disease(s) (5 points);
Nature of manuscript: original research (e.g., clinical practice; 10 points) or theoretical concept (literature review, hypothesis among others)? (5 points);
If the study had an evaluation component and/or used a study design to differentiate between study groups (5 points, respectively) in the manuscript.

The RA and the PI completed the shortlisted manuscript scoring for both teams. A cutoff score for relevance was set at 20 points.

Results are displayed in Figure 1. The RA obtained 1275 total hits with 215 manuscripts meeting the cutoff score for relevance. The librarian obtained 1232 hits, with 184 manuscripts meeting the relevant scoring criteria. Among the relevant manuscripts, there was a general overlap of 55 (16% of final literature capture) manuscripts between the two teams. The total unique literature capture was 344 manuscripts. The librarian's unique contribution to this literature capture (excluding the general overlap) was 129 manuscripts (37.5% of the final literature capture). Similarly, the unique contribution of the RA was 160 manuscripts (46.5%).

## Discussion

In this case study, we note two intertwined but critical aspects. First, the literature capture process by a researcher is a function of their training and experience. The hits obtained here are therefore unique to this researcher (A Tachble); other researchers may have higher or lower hit rates. This leads us to the process resulting in this variance. The librarian worked from the key words and used possible subject headings, related keywords and synonyms to refine a search process. Given that librarians are well acquainted with the anatomy of literature, they work from a very algorithmic process of deductive steps – from creating a very large catchment area, and reducing it by incorporating the exclusion criteria, and honing in on the critical mass by utilizing the inclusion criteria, to subject heading or other control vocabularies. This process of using documented search strategies is replicable between most librarians. Even a few researchers possibly follow these steps. Yet, we believe that most researchers will use databases to create citation lists – particularly databases like PubMed and Google Scholar. This is in essence browsing and utilizes snowballing techniques like perusing through the bibliography of identified hits. Interestingly, searching the term ‘Google Scholar’ in PubMed retrieves about 3926 results, as of August 2015. It would also seem that the 768 + 122 unique hits obtained by the researcher is a result of snowballing and being able to review its content as opposed to capturing it algorithmically; in other words, the manuscript may not be accurately represented by keywords and by extension of medical subject headings (MeSH) and other indexed terms.

The second important interrelated aspect we raise is time utilized by the researcher to compile the literature search. In this case study, the researcher took about 4 weeks to compile 1275 hits. Not all researchers have the luxury of an extended period of time to undertake a comprehensive literature review alone [8]. In contrast, the librarian would normally take 1–2 days to create and run algorithms, scan the results and rerun the algorithms with a few tweaks to improve or fine-tune the results.

We therefore contend that a literature review by a researcher alone may not be comprehensive using methods of browsing and snowballing. This is quite obvious in Figure 1 where 49 manuscripts that were identified by both teams were not included in the researchers’ final list, primarily because the full manuscript/source document could not be identified. Although their training in resource identification and retrieval is indispensable [9–11], the expectation that the literature search being the HSL's sole responsibility is – in our opinion – flawed. Our case study seems to indicate that in order to accomplish a literature search with due diligence, a deliberate researcher–HSL collaboration is necessary. Perhaps working in isolation from each other, then collating their findings could be a ‘best practice’ that produces a robust and comprehensive knowledge set.

Even though this is a single case study, and further research is strongly encouraged, from an ethics perspective, these results have important implications for clinical and institutional practices. Patients place their trust in clinicians to provide expert advice and care and in healthcare institutions to facilitate the delivery of good clinical practice through sound, well-informed guidelines. Clinical teams and institutions are legally and ethically bound to provide care that is partly knowledge-based, partly policy-based and partly skills-based. Each of these aspects has its relevant standard that is to be matched or surpassed by competent practitioners. The imperatives of evidence-based medicine are to: ensure that clinicians are aware of what would provide their patient populations with the best care and interventions possible, and inform institutional policy-makers about the relevant options in determining policy. Our findings suggest that the quality of the literature review yielded by the researcher–HSL partnership would satisfy these imperatives.

There are systemic implications too. One is with respect to informing development of clinical guidelines. The presentation of incomplete information may tend to generate an underinformed practice guideline that if implemented could result in causing avoidable harms (or perhaps even in misunderstood benefits). Another systemic implication is that by placing reliance on one form of literature review over the other, the direction of future research could become skewed. As the differences between results for the two searches show, literature blind spots arise. An author is not well placed to be able to report his or her own blind spots, but once published in a peer-reviewed forum, the article or report takes on a certain authority and lives a life of its own in future literature reviews. Any one viewpoint can only tell the story from its own perspective even when done with the good intention of generating evidence-based guidance for actual clinical decision-making. With this in mind, the advantage of having a multiple expertise-enriched literature review is obvious.

## Conclusion & future perspective

The results of this single case study demonstrate the advantages that researchers could have by not only involving professional librarians but also by becoming active participants in the literature search endeavor. The ethical implication of the absence of this process is large given the blind spots in the current way of doing literature reviews. For quality control purposes, we believe that that journal editors and peer-reviewers should have a checklist or a process in place to ensure that due diligence in the literature review has been done on any new submission.

**Executive summary**

A comprehensive literature review is the cornerstone of any scientific research endeavor.
Literature reviews are usually conducted by a researcher/team or by a health sciences librarian (HSL).
The literature capture processes used by researchers and HSLs working in isolation from each other – ‘browsing’ versus ‘algorithmic searching’ – are different, and both lead to blind spots.
To accomplish due diligence in a literature search – which has ethical and systemic implications – a deliberate researcher–HSL collaboration is necessary.
